# Gonadal Transcriptome Sequencing Analysis Reveals the Candidate Sex-Related Genes and Signaling Pathways in the East Asian Common Octopus, *Octopus sinensis*

**DOI:** 10.3390/genes15060682

**Published:** 2024-05-24

**Authors:** Fenghui Li, Siqing Chen, Tao Zhang, Luying Pan, Changlin Liu, Li Bian

**Affiliations:** 1 State Key Laboratory of Mariculture Biobreeding and Sustainable Goods, Yellow Sea Fisheries Research Institute, Chinese Academy of Fishery Sciences, Qingdao 266071, China; lifh@ysfri.ac.cn (F.L.); chensq@ysfri.ac.cn (S.C.); ply2208@stu.ouc.edu.cn (L.P.); liuchl@ysfri.ac.cn (C.L.); 2 Laboratory for Marine Fisheries Science and Food Production Processes, Laoshan Laboratory, Qingdao 266237, China; 3 Zhejiang Marine Fisheries Research Institute, Zhoushan 316021, China; zhangtao5729@163.com

**Keywords:** *Octopus sinensis*, gonadal transcriptome sequence, gene-expression profile, sex-related genes and pathways

## Abstract

The East Asian common octopus (*Octopus sinensis*) is an economically important species among cephalopods. This species exhibits a strict dioecious and allogamous reproductive strategy, along with a phenotypic sexual dimorphism, where the third right arm differentiates into hectocotylus in males. However, our understanding of the molecular mechanisms that underlie sex determination and differentiation in this species remains limited. In the present study, we surveyed gene-expression profiles in the immature male and female gonads of *O. sinensis* based on the RNA-seq, and a total of 47.83 Gb of high-quality data were generated. Compared with the testis, we identified 8302 differentially expressed genes (DEGs) in the ovary, of which 4459 genes were up-regulated and 3843 genes were down-regulated. Based on the GO enrichment, many GO terms related to sex differentiation were identified, such as sex differentiation (GO: 0007548), sexual reproduction (GO: 0019953) and male sex differentiation (GO: 0046661). A KEGG classification analysis identified three conserved signaling pathways that related to sex differentiation, including the Wnt signaling pathway, TGF-β signaling pathway and Notch signaling pathway. Additionally, 21 sex-related DEGs were selected, of which 13 DEGs were male-biased, including *Dmrt1*, *Foxn5*, *Foxj1*, *Sox30,* etc., and 8 DEGs were female-biased, including *Sox14*, *Nanos3*, *β-tubulin*, *Suh*, etc. Ten DEGs were used to verify the expression patterns in the testis and ovary using the RT-qPCR method, and the results showed that the expression level shown by RT-qPCR was consistent with that from the RNA-seq, which confirmed the reliability of the transcriptome data. The results presented in this study will not only contribute to our understanding of sex-formation mechanisms in *O. sinensis* but also provide the foundational information for further investigating the molecular mechanisms that underline its gonadal development and facilitate the sustainable development of octopus artificial breeding.

## 1. Introduction

Generally, sexual reproduction is defined as a reproductive process that generates new individuals based on the combination of two gametes (sperm and oocyte) from a male and a female, respectively [1,2]. It is one of the most pervasive and significant phenomena in biology, which has long fascinated biologists’ curiosity. Sex determination/differentiation, one of the most fundamental biological processes in sexual reproduction, has been investigated extensively in various organisms, revealing a diverse array of sex-determination mechanisms in both vertebrates and invertebrates [2]. However, to date, comprehensive knowledge of initiating male and female development remains constrained to a few well-established model species, such as *Caenorhabditis elegans*, *Drosophila melanogaster*, *Danio rerio*, *Mus musculus*, etc. [2,3,4,5]. These models, though informative, are insufficient to fully encapsulate the diversity of sex systems observed in nature. Therefore, understanding how sex is determined, differentiated and evolved requires more information from diverse taxa. Furthermore, understanding the mechanisms of animal sex differentiation is also crucial for artificial breeding and agricultural production. By mastering these mechanisms, we can more accurately control the sex ratio, optimize the population structure of cultured species and improve production efficiency [3]. 

Mollusca represents the second largest phylum after arthropods in the animal kingdom, containing around 20,000 living species that are widely distributed and often commercially important [6]. Mollusks exhibit a broad diversity in sexual systems and strategies, encompassing strict dioeciousness, hermaphroditism and even the capability of undergoing sex change [7], which makes them a beneficial clade for elucidating the intricate mechanisms of sex determination and evolution. The mechanisms of sex determination are remarkably diverse, yet they primarily fall into two broad categories: genetic sex determination (GSD) and environmental sex determination (ESD). Examples of environmental sex determination in mollusks are predominantly observed in sequential hermaphrodites, which allocate resources to either male or female functions based on environmental factors. For instance, the oyster has the capacity to switch sexes in response to cues such as nutrition and temperature [8,9]. The analyses of sex ratios and controlled crosses have suggested that genetic factors also play a crucial role in sex determination in mollusks [10,11]. However, the questions of whether the sex chromosomes exist and whether sex is determined by a single gene or polygenes in mollusks have confused biologists for a long time. Previous studies based on a karyotype analysis identified only a few species with sex chromosomes in gastropods and bivalves. These include the XX/XY system in *Littorina saxitalis*, *Atrina pectinata* and *Mulinia lateralis*; the XO/XX system in *Neotricula aperta*; the ZW/ZZ system in *Viviparus* spp.; as well as a multiple chromosomal sex-determination system (male: XY_1_Y_2_) in *Carinaria japonica* [12,13,14], which indicates that the sex chromosome seems to exist in mollusks with a strict dioecious system. Nevertheless, as a group that originated anciently, a majority of mollusks, even those that are dioecious, have not evolved sex chromosomes [15], which makes karyotype analysis inefficient for sex-determination investigations in mollusks. Recently, based on many molecular biology techniques, such as Amplified fragment length polymorphism (AFLP), Restriction-site-associated DNA sequencing (RAD-seq), genomic sequencing and re-sequencing, transcriptome sequencing, etc., the genetic factors of sex determination have been studied in greater detail. Up to now, not only have many sex-related markers, sex-related genes and pathways and quantitative trait loci (QTLs) for sex been identified [16,17,18,19], but some novel insights into the sex-determination mechanism in mollusks were provided. For example, the research conducted by Yue et al. [20] based on the RAD-seq showed no evidence for sex chromosomes or single-locus models for *Crassostrea gigas* primary sex determination, and they proposed a sex-determination hypothesis involving multiple genetic factors. 

The genes involved in the sex-determination/differentiation process are always expressed in a sexually dimorphic manner. Therefore, transcriptome sequencing technology has been used as an important tool for identifying sex-determination and -differentiation genes in many kinds of animals, including mollusks. Currently, a number of sex-related genes have been identified from diverse mollusks based on a gonadal transcriptome analysis. These include transcription factor *Sox2* (*Sox2*), forkhead box protein Z (*Foxz*), heat shock transcription factor, Y-linked (*Hsfy*), forkhead box protein L2 (*Foxl2*) and transcription factor HES-1 (*Hes1*) in dwarf surf clam (*M. lateralis*) [21]; *Foxl2*, beta-catenin (*β-catenin*) and sex-lethal (*Sxl*) in blood clam (*Tegillarca granosa*) [22]; *FoxL2* and doublesex- and mab-3-related transcription factor A2 (*Dmrta2*) in Pacific abalone (*Haliotis discus hannai*) [23]; transcription factor *SoxH* (*SoxH*), *FoxL2*, doublesex (*Dsx*), feminization (*Fem*), GATA binding protein 4 (*Gata4*), wnt family member 4 (*Wnt4*) and *β-catenin* in Pacific oyster (*C. gigas*) [24,25]; testis-specific serine/threonine-protein kinase 1 (*Tssk1*), testis-specific serine/threonine-protein kinase 4 (*Tssk4*), testis-specific serine/threonine-protein kinase 5 (*Tssk5*), doublesex- and mab-3-related transcription factor 1 (*Dmrt1*), sperm protein 17 (*Sp17*) and feminization-1 (*Fem1*) in fluted giant clam (*Tridacna squamosa*) [26]; *DmrtA2*, transcription factor *Sox9* (*Sox9*), *Fem-1b*, *Fem-1c*, vitellogenin (*Vg*), cytochrome P450 family 17 subfamily A member 1 (*Cyp17a1*) and spermatogenesis- and oogenesis-specific basic Helix-Loop-Helix 2 (*Sohlh2*) in razor clam (*Sinonovacula constricta*) [27]; and *Dmrt1*, transcription factor *Sox30* (*Sox30*), testis-specific serine/threonine-protein kinase (*Tssk*), *Gata1* and *Vg* in yesso scallop (*Patinopecten yessoensis*) [17]. The sex-determination genes in vertebrates, such as *Foxl2*, *Dmrt1* and *sry*, were also identified in mollusks, which suggests that the sex-determination mechanisms in animals may be conserved. Among these sex-related genes, Nanos homolog (*Nanos*), *Vasa* and P-Element-induced wimpy testis (*Piwi*) are critically important for germ cell development [28,29,30], and *Foxl2*, *Dmrt1*, *Fem1*, *SoxH* and *Tssk1* play pivotal roles in sex maintenance or gametogenesis [11,26,31]. Till now, the knowledge regarding the molecular mechanisms of sex determination/differentiation in mollusks mainly focuses on the species within bivalves that are widely cultured and economically important. While cephalopods, such as cuttlefish, squid and octopus, also have a high economic value, information on them is still scarce. 

Unlike most mollusks, such as bivalves, octopuses in the class Cephalopoda present a strict dioecious and allogamous reproductive strategy and phenotypic sexual dimorphism that the third right arm differentiates into hectocotylus in males [32]. Recently, the comparison of male and female genomic sequences in the California two-spot octopus (*Octopus bimaculoides*) has uncovered a Z sex chromosome, which confirmed that the octopus may employ ZZ/ZO (males/females) as the sex-determination system [33]. However, how sex is determined/differentiated and evolved in octopuses is still limited. The East Asian common octopus, *O. sinensis* (d’Orbigny, 1841), lives mainly in shallow temperate waters of the western North Pacific oceans, particularly in the coastal regions of South Korea, China and Japan [32]. Previous studies mainly focused on artificial breeding [34,35]. The availability of the *O. sinensis* genome [36] provides the opportunity to investigate these complex molecular mechanisms in the sex-formation process of *O. sinensis*. In the present study, we surveyed the gene-expression profiles of immature male and female gonads of *O. sinensis* based on the bulk RNA-seq. The results presented in this study will not only contribute to our understanding of sex-formation mechanisms in *O. sinensis*, but they will also provide the fundamental basis for further investigating the molecular mechanisms underlying its gonadal development and facilitate the sustainable development of octopus artificial breeding.

## 2. Materials and Methods

### 2.1. Experimental Octopus

A total of 21 wild *O. sinensis* were captured from the littoral waters of Zhoushan, Zhejiang Province, China (29°53′36.98″ N, 122°18′29.01″ E) on 15 February 2022. Male and female individuals were separated and cultured for one week before sample collection. Subsequently, the gonadal tissues of all individuals were dissected. Some of these tissues were immediately frozen in liquid nitrogen and then transferred to a −80 °C freezer for RNA extraction, while the remaining tissues were fixed in Bouin’s solution for further histological analysis. Before gonad dissection, the octopuses were anesthetized in 20% anhydrous ethanol prepared using filtered seawater. All of the animal experiments in this study were approved by the Animal Care and Use Committee of the Chinese Academy of Fishery Sciences (IACUC-2022-03).

### 2.2. Histological Analysis and Samples Collection

The gonadal tissues were fixed in Bouin’s solution for 24 h and then embedded in paraffin after the process of dehydration and transparency. The tissue blocks were then cut into 5 µm continuous slices on a rotary microtome (Leica, Wetzlar, Germany). After being stained with hematoxylin and eosin (HE), the slices were observed under an Eclipse E600 research microscope (Nikon, Tokyo, Japan).

The immature gonadal tissues were selected based on a histological analysis. Namely, three males (OSIMT1-3) with a mean body weight of 268.51 ± 13.06 g, mean body length of 39.65 ± 3.35 cm and gonadosomatic index (GSI) of 0.31 ± 0.24 and three females (OSIFO1-3) with a mean body weight of 182.31 ± 20.29 g, mean body length of 40.4 ± 2.43 cm and GSI of 0.55 ± 0.22 were selected for RNA-seq. Three males and three females whose GSI were similar to that of RNA-seq samples were selected for Real-Time Quantitative PCR (RT-qPCR) verification.

### 2.3. RNA Library Construction, Sequencing and Reference-Based Assembly

Total RNA was isolated using Trizol reagent (Invitrogen, Carlsbad, CA, USA) following the manufacturer’s instructions. RNA concentration, purity and integrity were measured using NanoDrop 2000 (Thermo Fisher Scientific, Wilmington, DE, USA) and the RNA Nano 6000 Assay Kit of the Agilent Bioanalyzer 2100 system (Agilent Technologies, Santa Clara, CA, USA). The high-quality RNA samples were sent to the Biomarker Technologies Corporation (Beijing, China) for cDNA library construction and sequencing. cDNA libraries were constructed using the NEBNext^®^ Ultra™ RNA Library Prep Kit for Illumina (New England Biolabs, Ipswich, MA, USA) following the manufacturer’s protocol. The quality, insert size and concentration of the cDNA libraries were assessed using agarose gel electrophoresis, Agilent 2100 (Agilent Technologies, Santa Clara, CA, USA) and a Qubit^®^ 3.0 fluorometer (Thermo Fisher Scientific, Waltham, MA, USA), respectively. Six well-prepared RNA-seq libraries were sequenced on an Illumina HiSeq X platform for paired-end sequencing, and 150 bp paired-end reads were generated.

Initially, the raw data underwent processing using in-house Perl scripts, where clean data were generated by eliminating reads that contained adapter sequences, poly-N and reads of low quality. Subsequently, TopHat2 [37] tools were used to map the clean reads of each sample to reference the genome of *O. sinensis* [GCF_006345805.1]. The mapped reads were assembled using StringTie 2.2.3 software [38], and the novel genes were identified based on the comparison between assembled transcripts and reference genome annotation. For novel gene annotation, the novel transcripts were aligned to different databases, including Swiss-Prot, NR (the NCBI nonredundant protein database), GO (Gene Ontology) and KEGG (the Kyoto Encyclopedia of Genes and Genomes) using BLAST 2.15.0 software [39], and the KOBAS 2.0 and HMMER 3.3.2 software were used for the Pfam annotation of the novel genes [40,41].

### 2.4. Gene-Expression Analysis and Sample Relationship Analysis

The RSEM v1.2.26 software was used to count the number of reads mapped to each gene [42]. The relative expression level of each gene was calculated based on the Fragments per Kilobase of transcript per Million fragments mapped (FPKM) using the StringTie method [43,44]. Afterward, the reconstruction of the transcript assemblies was carried out using the reference genome annotation-based transcripts assembly program within the Cufflinks 0.7.0 software package [45], aiming to obtain a comprehensive set of transcripts for further differential analysis.

In order to study the global transcriptomic differences and correlations among samples from the two sexes, a principal component analysis (PCA) and a heat map were constructed to evaluate the repeatability between samples based on the FPKM values from all expressed genes in each sample. Both PCA and heatmap construction were performed using BMKCloud (www.biocloud.net, accessed on 1 October 2015). 

### 2.5. DEGs Identification and Function Enrichment

The DEGs were identified using the DESeq2 R package [44]. DEGs were defined as |Log2FoldChange| > 2 with a false-discovery rate (FDR) < 0.01. The transcriptional profile variations between the two sexes were assessed by DEG union, and R scripts were used to generate a heat map of the DEGs. A Gene Ontology (GO) enrichment analysis of the differentially expressed genes (DEGs) was implemented by the GOseq R packages based on a Wallenius noncentral hyper-geometric distribution [46], which can adjust for gene length bias in DEGs. KEGG is a database resource for understanding high-level functions and utilities of the biological system, such as the cell, the organism and the ecosystem, from molecular-level information, especially large-scale molecular datasets generated by genome sequencing and other high-throughput experimental technologies. We used KOBAS 3.0 software to test the statistical enrichment of differential expression genes in KEGG pathways.

### 2.6. Real-Time Quantitative PCR (RT-qPCR) Verification

The RT-qPCR method was employed to validate the transcriptome sequencing data. The total RNA was extracted from the samples selected for RT-qPCR using Trizol reagent (Invitrogen, Carlsbad, CA, USA). The concentration, purity and integrity of the total RNA were measured using NanoDrop 2000 (Thermo Fisher Scientific Wilmington, DE, USA) and agarose gel electrophoresis methods. cDNA was synthesized using a PrimeScript™ RT reagent Kit with a gDNA Eraser (Takara, Ohtsu, Japan). RT-qPCR was performed using SYBR^®^ Premix Ex Taq (Takara, Ohtsu, Japan) according to the manufacturer’s instructions on a StepOnePlusTM Real-Time PCR system (Applied Biosystems, FosterCity, CA, USA) in 20 μL reactions. The PCR amplification procedure was carried out at 95 °C for 90 s, followed by 40 cycles at 95 °C for 5 s, 60 °C for 15 s and 72 °C for 20 s. The β-actin gene was selected as the endogenous reference gene. Ten DEGs were selected for RT-qPCR verification whose primer sequences were designed using Primer 5.0 software. The 10 pairs of primers are shown in Table 1. The gene relative expression level was calculated with the 2^−ΔΔCt^ method [47]. A one-way analysis of variance in SPSS 20.0 (IBM, Armonk, NY, USA) was used for statistical analysis, and *p* < 0.05 was defined as a significant difference. The FPKM value generated from RNA-seq and the gene relative expression data generated from RT-qPCR were used for graphical presentations.

## 3. Results

### 3.1. Histological Structure of Immature Gonads

**Figure 1:**
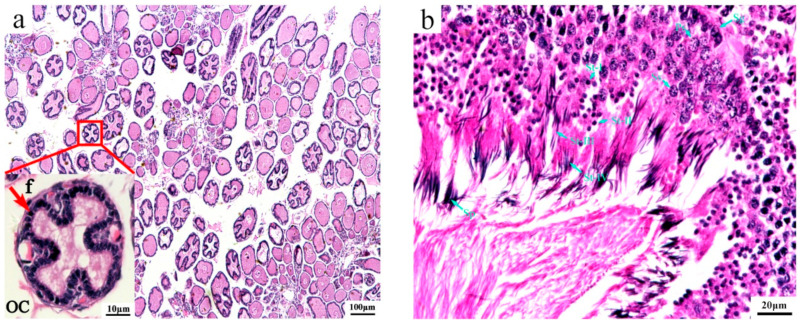
Histological analysis of gonads of *O. sinensis.* (**a**) Immature ovary, f: follicle cell, oc: oocyte; (**b**) immature testis, Sg: spermatogonium, Ps: primary spermatogonia, Ss: secondary spermatogonia, St-I: spermatid stage I, St-II: spermatid stage II, St-III: spermatid stage III, St-IV: spermatid stage V, Sp: sperm.

To better understand the mechanisms of sex determination/differentiation in *O. sinensis*, we aimed to use immature gonads as research objects. To accurately determine the developmental stage of the gonads of the wild *O. sinensis* captured, we employed a histological analysis. The cytological characteristics of the immature gonads are shown in Figure 1. At this stage, the ovary (Figure 1a) was filled with spherical or ovoidal oocytes with a diameter of around 50–110 µm. Oocytes were surrounded by a double layer of follicle cells: the outer layer was flattened, and the inner layer was cuboidal. Additionally, the follicle cells of some oocytes started to enfold into the ooplasm. The ooplasm was compact in oocytes without folds and was vacuolated in the oocytes with folds. The testis (Figure 1b) was occupied mainly by spermatogonium, primary spermatogonia, secondary spermatogonia and spermatids (I-V), and only a small number of mature sperms were observed.

### 3.2. Overall Transcriptome and Sequencing Data

Based on the histological analysis, six cDNA libraries named OSIFO1-3 and OSIMT1-3 were constructed for RNA-seq, and the data-processing results are shown in Table 2. After quality control, a total of 47.83 Gb of clean data were generated, including 320,462,646 clean reads. The clean data for each sample were at least 6.71 Gb. The GC content and Q30 of each sample were above 39.17 and 92.61%, respectively. The clean reads of each sample that successfully mapped to the reference genome were 41,856,883, 47,116,323, 41,546,433, 57,439,795, 62,552,327 and 48,335,480, with a mapped ratio ranging from 92.35 to 94.42%, a unique mapped ratio ranging from 89.41 to 90.46% and a multiple mapped ratio ranging from 2.80 to 3.96%. Furthermore, 63.68 to 75.78%, 4.32 to 5.96% and 18.90 to 30.36% of the reads were mapped to the exon, intron and intergenic regions of the reference genome, respectively (Supplementary Table S1).

### 3.3. Different Expression Gene Identification

**Figure 2:**
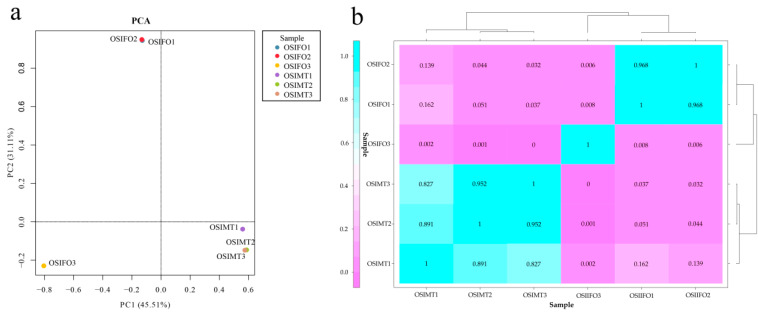
Correlation analysis between repeated samples. (**a**) Principal component analysis reveals strong clustering associated with sex (PC1 accounted for 45.51% of the variance). (**b**) Heat maps of the repeated samples. Blue signifies a strong correlation while pink indicates a weak correlation.

To improve the data accuracy and repeatability, the PCA and a sample relationship heat map were constructed based on the FPKM values of all expressed genes (Figure 2). The PCA showed strong clustering associated with sex, except for the sample OSIFO3 (Figure 2a). PC1 accounted for 45.51% of the variance and revealed strong clustering associated with sex. However, PC2 accounted for only 31.11% of the variance, which showed that repeated samples were not well clustered, especially the sample OSIFO3, which indicated that the development stage of the OSIFO3 may be different from the OSIFO1 and OSIFO2. In the sample relationship analysis, the heat map also showed a similar result with PCA (Figure 2b). The Pearson’s correlation coefficient (r2) of OSIFO1 and OSIFO3 and OSIFO2 and OSIFO3 was 0.008 and 0.006, respectively, which showed an extremely low coefficient. In the subsequent analysis, the RNA-seq data of the sample OSIFO3 were excluded. Then, 18,184 known protein-coding genes were identified by mapping the clean data to the reference genome. Additionally, a total of 8062 novel genes were identified, of which 5565 novel genes were functionally annotated (Supplementary Table S2). 

**Figure 3:**
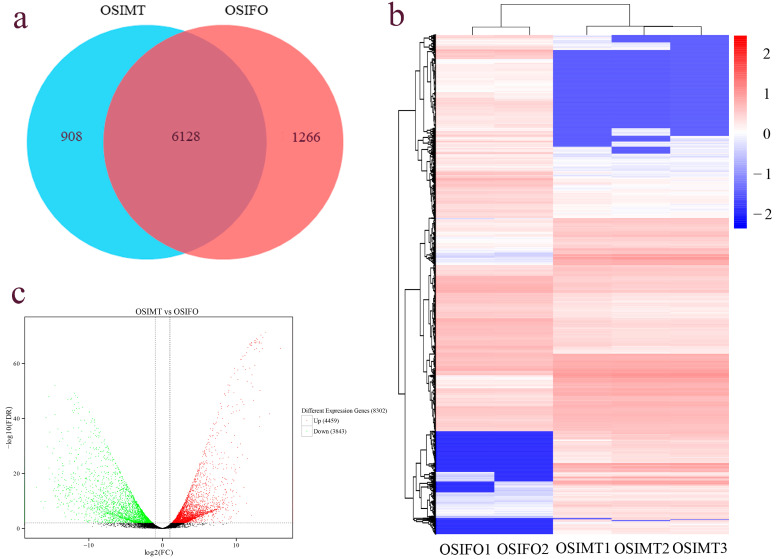
The statistics of different expression genes in the gonad of *O. sinensis*. (**a**) The Venn diagram; (**b**) volcano map; (**c**) heat map.

In the differential expression analysis, 8302 differentially expressed genes were identified in the female and male gonad groups. Compared with the male gonad group, 4459 DEGs were significantly up-regulated and 3843 DEGs were significantly down-regulated in the female gonad group (Figure 3b). Among all DEGs, 6128 DEGs were expressed in both the female and male gonad groups, 908 DEGs were only expressed in the male gonad group and 1266 DEGs were only expressed in the female gonad group (Figure 3a). The heat map constructed based on the FPKM values of all DEGs showed that five samples were clustered into two groups, and the male and female gonad samples could be evidently distinguished (Figure 3c). 

### 3.4. Functional Annotation, Classification and Enrichment Analysis of DEG

**Figure 4:**
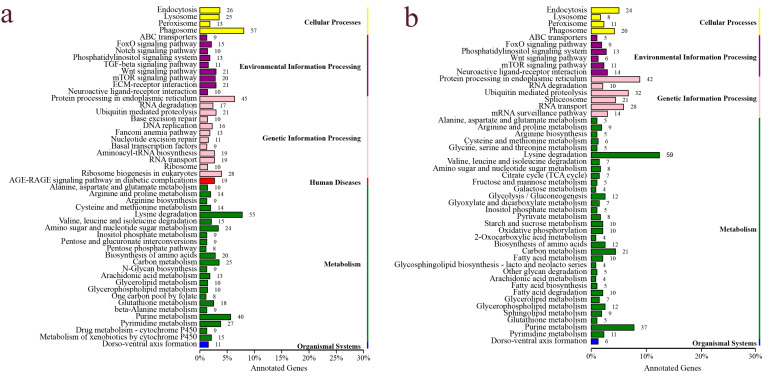
The KEGG classifications of the DEGs in the testis and ovary of the *O. sinensis*. (**a**) Up-regulated genes, (**b**) down-regulated genes.

**Figure 5:**
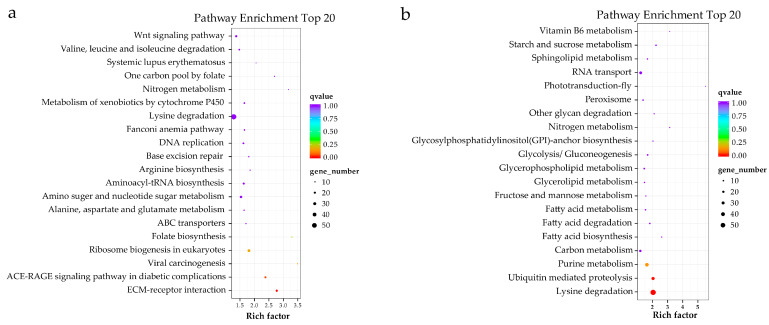
The top 20 KEGG pathways enriched by DEGs in ovary and testis of *O. sinensis*. (**a**) The up-regulated DEGs; (**b**) the down-regulated DEGs.

**Figure 6:**
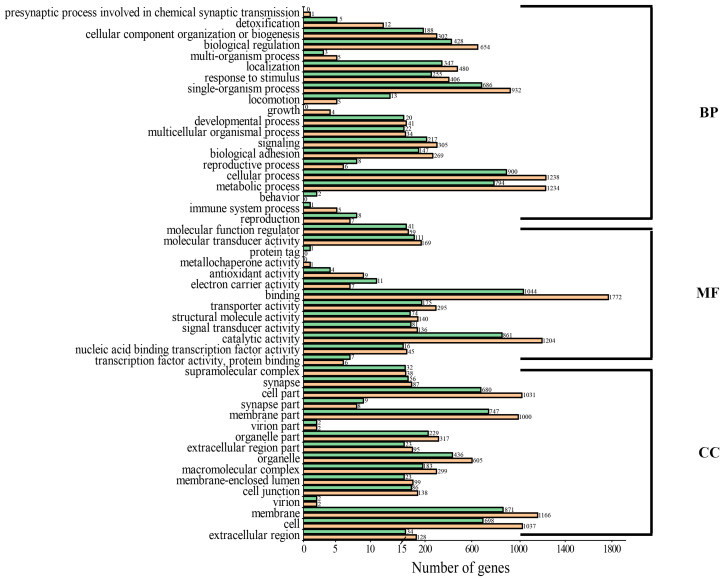
GO annotation and classification of DEGs in the gonad of *O. sinensis*. BP: biological process, MF: molecular function, CC: cellular component, number: the number of genes in the corresponding secondary pathway, green: down-regulated genes, yellow: up-regulated genes.

By aligning to different functional databases, including GO, KEGG, Swiss-Prot and NR databases by BLASTX, a total of 7471 DEGs were functionally annotated, accounting for 90% of all DEGs identified (Table S3). Based on the KEGG annotation, a total of 1185 DEGs were annotated to KEGG signaling pathways, including 708 up-regulated DEGs and 477 down-regulated DEGs. Up-regulated DEGs were classified into 50 signaling pathways of six branches. The top four pathways were “Phagosome”, “Protein processing in endoplasmic reticulum”, “Lysine degradation” and “Purine metabolism”, with 57, 45, 55 and 40 DEGs, respectively (Figure 4a). The down-regulated DEGs were classified into 49 signaling pathways, with the top four pathways being “Protein processing in endoplasmic reticulum”, “Lysine degradation”, “Purine metabolism” and “Endocytosis”, with 42, 59, 37 and 20 DEGs, respectively (Figure 4b). Based on the KEGG enrichment analysis of the DEGs, two pathways, including the “AGE-RAGE signaling pathway in diabetic complications” and “EMC-receptor interaction”, were significantly enriched in the ovary (Figure 5a), and two pathways, including “Ubiquitin mediated proteolysis” and “Lysine degradation”, were significantly enriched in the testis (Figure 5b). Notably, several classical signaling pathways related to sex determination/differentiation in animals were identified, such as the Wnt signaling pathway, TGF-β signaling pathway, Notch signaling pathway, etc. (for the related genes included in these signaling pathways, see Supplementary Tables S4–S6). According to the GO database annotation, a total of 4845 DEGs, including 2914 up-regulated DEGs and 1931 down-regulated DEGs, could be classified into 49 subcategories in three main categories: biological process (BP), cellular component (CC) and molecular function (MF) (Figure 6). In the BP, the GO terms of the cellular process, metabolic process and single-organism process were the top three subcategories. In CC, the membrane, cell, cell part and membrane part were the four most dominant GO terms. For the MF, the predominant GO terms were binding and catalytic activity. Additionally, in the GO enrichment analysis, 11 GO terms including nine DEGs related to sex determination/differentiation were classified (Table 3). Among the 11 GO terms, the sex differentiation (GO:0007548) including three genes was significantly enriched (*p* < 0.05) (Supplementary Table S7).

### 3.5. Identification of Sex-Related Genes in O. sinensis

**Figure 7:**
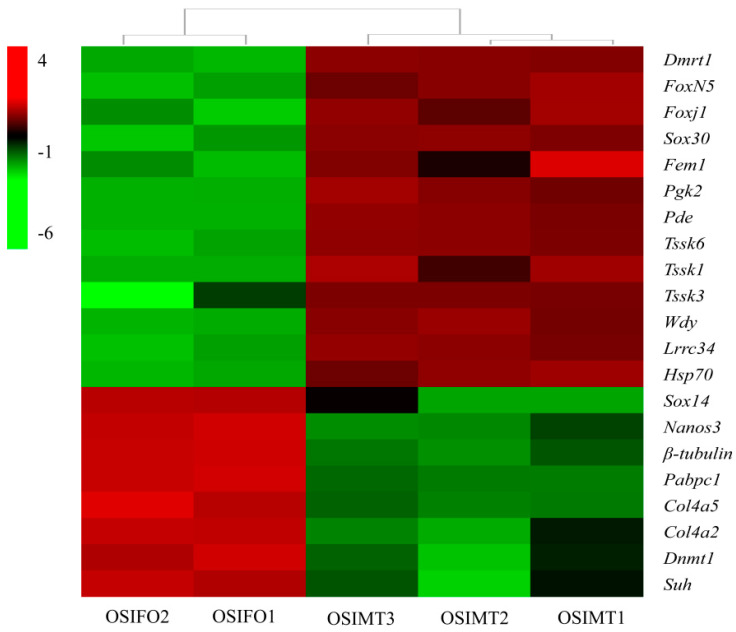
A heatmap reveals the different expression patterns of sex-related genes in the immature male and female gonads of *O. sinensis*.

Based on the overall analysis of gene-expression profiles, 21 sex-related genes were identified in the gonad of *O. sinensis* (Table 4, Figure 7). A total of 13 DEGs were male-biased, including *Dmrt1*, Forkhead box protein N-5-like (*Foxn5*), forkhead box protein J1-B-like (*Foxj1*), *Sox30*, *Fem1*, phosphoglycerate kinase 2-like (*Pgk2*), cGMP-specific 3′,5′-cyclic phosphodiesterase-like (*Pde*), testis-specific serine/threonine-protein kinase 6-like (*Tssk6*), *Tssk1*, testis-specific serine/threonine-protein kinase 3-like (*Tssk3*), WD repeat-containing protein on Y (*Wdy*), chromosome leucine-rich repeat-containing protein 34-like and heat shock protein 70 B2-like (*Lrrc34*). Others were female-biased, including protein nanos 3 (*Nanos3*), heat shock protein 70 B2-like (*Hsp70*), tubulin beta chain-like (*β-tubulin*), polyadenylate-binding protein (*Pabpc1*), collagen alpha-5(IV) chain-like (*Col4a5*), collagen alpha-2(IV) chain-like (*Col4a2*), DNA (cytosine-5)-methyltransferase 1-like (*Dnmt1*) and recombining binding protein suppressor of hairless-like (*Suh*). All these selected DEGs were significantly differentially expressed in the testis and ovary (FDR ≤ 0.01), which indicated that they play an important role in sex determination/differentiation and reproduction in *O. sinensis*.

### 3.6. Real-Time Quantitative PCR (RT-qPCR) Verification

**Figure 8:**
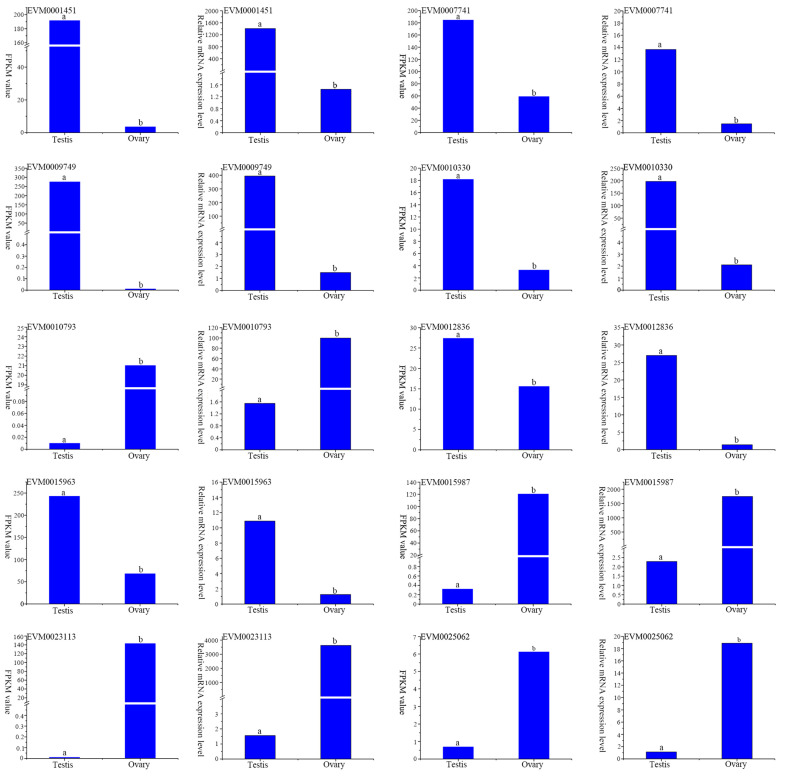
Verification of the gene-expression patterns in the transcriptome analysis using RT-qPCR method. Groups denoted with different letters exhibited statistically significant differences (*p* < 0.05).

To validate the accuracy of the transcriptome data, 10 DEGs were randomly selected for RT-qPCR analysis. The results showed that the expression profiles of all genes indicated by RT-qPCR analysis were similar to those indicated by RNA-seq (Figure 8), indicating the reliability and accuracy of the transcriptome expression analysis.

## 4. Discussion

*O. sinensis* shows a strict dioecious and allogamous reproductive strategy and sexually dimorphic phenotypes, in which the third right arm of males differentiates into a hectocotylized arm that is used for sperm transportation. These unique characteristics make it an interesting species for investigating the mechanisms of sex formation and evolution in Mollusca. Besides, given the high commercial value of *O. sinensis*, studies on the molecular mechanisms of sex determination/differentiation and gonad development are necessary. Previous studies have increased our understanding of sex-determination/differentiation mechanisms in mollusks and started to indicate the key genes and signaling pathways underlying this complex biological process. 

### 4.1. Overall Characteristics of the Transcriptome Data 

With the improvement in next-generation sequencing technology and bioinformatics analysis methods, transcriptomics has been widely used to profile the expression of genes regulating the development and response to diverse environmental stress in mollusks [48,49,50,51]. In the present study, a total of 47.83 Gb of clean data, including 320,462,646 clean reads, were generated from the immature gonads of *O. sinensis* using RNA-seq. The Q30 was above 93% and the reads were mapped to the reference genome with an above 92% probability (Table 2 and Table S1). All these characteristics of the transcriptome data indicated that the sequencing was efficient and of high quality. Additionally, the consistency of the gene-expression level between RT-qPCR and RNA-seq confirmed that the transcriptome data were accurate and reliable (Figure 8). 

### 4.2. Signaling Pathways and GO Terms Related to Sex-Determination/differentiation Process

By comparing the transcriptomes of male and female gonads of *O. sinensis*, we identified 8302 DEGs, including 4459 up-regulated DEGs and 3843 down-regulated DEGs (testis vs. ovary). In total, 7471 DEGs were functionally annotated, accounting for 90% of all DEGs identified (Table S3). The KEGG classification identified several signaling pathways, such as the Wnt signaling pathway, TGF-β signaling pathway and Notch signaling pathway (Figure 4). In mammals, the classical Wnt signaling pathway is β-catenin-mediated. In this process, the Rspol and Wnt4 co-activate the expression of β-catenin to inhibit Sox9/Fgf9 (male-related genes) expression and promote follistatin (*Fst*) expression to determine the ovary fate [52]. Additionally, the Wnt signaling pathway also plays an important role in maintaining germ cell development in the testis and ovary [53,54,55], which may be the reason why it was classified in both sexes of *O. sinensis*. The TGF-β signaling pathway can regulate gonad differentiation by regulating the number of germ cells and expression levels of aromatase genes. In medaka, the *Amhr2* male mutant exhibited remarkable phenotypic abnormalities, including sex reversal and proliferation of the germ cells [56]. The TGF-β signaling pathway is also essential for ovary development. The TGF-β molecules, such as *Activins*, *Inhibins*, bone morphogenetic protein 7 (*Bmp7*) and growth differentiation factor 9 (*Gdf9*), play important roles in the ovary-maturation process in *Micropterus salmoides* based on the transcriptome analyses [57]. In our study, the TGF-β signaling pathway was only classified in the ovary of *O. sinensis*, indicating that the TGF-β molecules might be involved in the regulation of ovary differentiation and development. The Notch signaling pathway is one of the important pathways that is involved in some basic biological processes, including cell proliferation stem cell maintenance and differentiation during embryonic and adult development in animals [58]. In mammals, the Notch signaling pathway can regulate ovary differentiation and development. The suppression of Notch signaling in the neonatal mouse ovary decreased primordial follicle formation, and the function of the testes declined in male mice overexpressing notch homolog 1 (*Notch1*) [59,60,61]. In mollusks, the Notch signaling pathway was also proved to be involved in determining the sex fate in *Hyriopsis cumingii*, *Crassostrea hongkongensis* and *Mytilus unguiculatus* [62,63,64]. In the current transcriptome study of *O. sinensis*, the classification of these pathways suggests their potential involvement in the regulation of sex determination/differentiation and gonad development in this species. 

### 4.3. Key Sex-Related Differentially Expressed Genes

Based on the transcriptome analysis, at least 21 DEGs involved in sex determination/differentiation were identified. These genes included *Dmrt1*, *Foxn5*, *Foxj1*, *Sox14*, *Sox30*, *Fem1*, *Tssk6*, *Tssk1*, *Tssk3* and other potential candidates that have been reported previously in vertebrates and in mollusks [21,22,23,24,25,26,27]. 

*Dmrt1* belongs to the DMRT gene family, which is a well-known gene related to sex determination in vertebrates [65]. In zebrafish, the expression level of *Dmrt1* in the testis is significantly higher than that in the ovary, and the ovary development recovered in the male mutants lacking this gene [66]. In mollusks, Naimi et al. [67] cloned the *Dmrt1* gene for the first time and named it *Cg-DMl*, and they found that the expression profile is similar to that in vertebrates, so it was speculated that this gene was also involved in the sex-determination process in oysters. Subsequently, *Dmrt1* was cloned successively from *H. cumingii*, *Hyriopsis schlegelii* and *P. yessoensis,* and researchers found a similar expression profile with oysters [68,69,70]. The study conducted by Zhou et al. [17] proposed that *Dmrt1* plays a key role in *P. yessoensis* sex determination. The expression of *Dmrt1* can activate male-related genes such as *Sox30*, leucine-rich repeat-containing protein (*Lrr*), stabilizer of axonemal microtubules (*MTs*), WD repeat-containing protein on Y chromosome-like (*WD rcp)*, *Tssk3* and *Pde* to determine the male fate. In our study, *Dmrt1* in *O. sinensis* presented a similar expression profile to that in other mollusks, which indicated that it might participate in the sex-determination process.

The fox gene family encodes a series of transcription factors that contain the forkhead domain, which is involved in many biological processes, including embryogenesis, apoptosis, immune response, metabolic processes, sex determination and gonad development [71,72]. In mollusks, *Foxl2* was presumed to be a key candidate gene for sex determination and differentiation. It had been identified in *Chlamys farreri* [73], *C. gigas* [25], *P. yessoensis* [31] and *P. margaritifera* [11] and presented an ovary-biased expression profile, so it was supposed to be a master gene determining the ovary fate. However, we did not find the *Foxl2* gene in our study. Whether the function of *Foxl2* in sex determination/differentiation was conserved in mollusks still requires further elucidation. *Foxn5* (*Foxr1*) is a novel gene that has been recently identified and is supposed to be involved in sex determination. Interestingly, this gene exhibits a different expression profile in different species. In mammals, *Foxn5* is male-biased, while it is female-biased in fish [74,75]. In our study, based on GO enrichment, the *Foxn5* (EVM0026626) was annotated to the GO term of sex determination/differentiation (Table 3). The expression level in the testis was significantly higher than that in the ovary (FDR ≤ 0.05), which is similar to that in oysters [25]. All this evidence indicates that *Foxn5* may contribute to male differentiation and development in mollusks. 

The SOX superfamily encodes a series of proteins with one or more conserved HMG (high mobility group) domains. Since the *Sry* (sex-determining region on the Y chromosome) gene was cloned for the first time from mammals, more than 40 members have been identified and are involved in multiple biological processes, including sex determination/differentiation, testis development and male fertility maintenance [76]. For example, *Sry* cooperates with *Sox9* to activate the *Dmrt1*-mediated male signaling pathway to regulate male sex determination in mammals [77]. *Sox3* plays crucial roles in gametogenesis, sex determination and gonad differentiation in fish [78]. *Sox30* is the only member of the SoxH subfamily that is considered to be involved in spermatogonial differentiation and spermatogenesis in vertebrates [79]. In mollusks, *Sox30* has been identified from the gonads of several species, such as *C. gigas* [25], *Ruditapes philippinarum* [80] and *P. yessoensis* [12]. The expression of *Sox30* in the testis of all these species was significantly higher than that in the ovary, which is consistent with our results. Therefore, we suppose that *Sox30* is a candidate gene for sex determination and differentiation in *O. sinensis*. Another sox gene we identified from the *O. sinensis* gonads was *Sox14,* which was expressed in both the testis and ovary but presented a female-biased expression pattern, which is similar to that in crustaceans [81]. However, in *H. cumingii*, a freshwater mollusk, the *Sox14* gene exhibited an opposite expression pattern, with significantly higher expression levels in the testis compared to the ovary [82]. Therefore, the specific function of Sox14 in the gonads of *O. sinensis* requires further study.

The fem genes including *Fem1*, *Fem2* and *Fem3* are key genes that regulate sex determination/differentiation and gonadal development. In *C. elegans*, the expression of fem genes can induce *Mab-3* expression and determine the male fate [83]. *Fem1* contains three homologs (*Fem1a*, *Fem1b* and *Fem1c*) and was reported to be related to sex determination in humans and house mice [84]. In mollusks, based on a transcriptome analysis, the *Fem1* homologs were found in *P. yessonsis*, *S. constricta* and *P. margaritifera*. The *Fem1* genes in the immature *S. constricta* and *P. margaritifera* presented a male-biased expression pattern, while *Fem1c* in mature *P. yessonsis* was female-biased [11,27,85]. Yu et al. [86] also found that the expression levels of *Fem1* genes in males were significantly higher than those in females in the early stage of *Litopeneaus vannamei,* and there was a shift toward higher expression in females during the mysis and post-larval stages; thus, they concluded that the *Fem1* genes may contribute to sex differentiation and ovary development in shrimps. Based on the RNA-seq, we observed a significantly higher expression level of *Fem1* in the male gonads of *O. sinensis*, which suggested its function in sex determination and differentiation.

*Tssk1*, *Tssk3* and *Tssk6* were identified from the gonad transcriptome data and presented a male-specific expression pattern that is consistent with that in *S. constricta*, *P. yessoensis* and *T. squamosa*, which indicated their function in male gonad development [12,26,27]. The testis-specific serine/threonine-protein kinase (*Tssk*) genes encode the testis-specific serine kinase proteins that are composed of six members: Tssk1 through a Quantitative PCR analysis and immunolocalization showed that Tssk1 and Tssk6 are present in the mouse testis and in mouse and human sperm, but not Tssk3, yet Tssk3 mRNA was expressed in spermatids [87], which strongly suggested that Tssks have important roles in germ cell differentiation and possibly sperm function.

## 5. Conclusions

In conclusion, the cDNA library of immature male and female gonads was constructed for the first time. In total, 47.83Gb of clean data were obtained using the Illumina sequencing platform. Additionally, the whole gene-expression profile was explored based on a bioinformatics analysis. The RT-qPCR verification results indicated that accurate and reliable transcriptome data were obtained. Importantly, multiple sex-related genes, such as *Dmrt1*, *Foxn5*, *Sox30*, etc., and pathways, such as the Wnt signaling pathway, TGF-β signaling pathway and Notch signaling pathway were identified and involved in sex determination/differentiation in *O. sinensis*. The results presented in this study will not only contribute to our understanding of sex-formation mechanisms in *O. sinensis*, but they will also provide the foundational basis for further investigating the molecular mechanisms underlying its gonadal development and facilitate the sustainable development of octopus artificial breeding.

## Figures and Tables

**Table 1:** Gene primers used for RT-qPCR.

Gene ID	Forward Primer	Reverse Primer
β-actin	TGATGGCCAAGTTATCACCA	TGATGGCCAAGTTATCACCA
EVM0015963	AACCTGCTCTTTGCTCTGCAT	CAATGCAGCTGGCTACTGGAC
EVM0007741	TCCCCTTCTAATCAGACCGC	AAGTCAGGAAGGAACTGCTAAC
EVM0010793	CCGTGGTTATGGACAGACTTC	CCCGTTCCTCTTCACTCTTAT
EVM0025062	AGCCGAGCGAACTACAGTACCTC	GGCTGACTGACTTGTACCTCTGC
EVM0010330	ACCGTCCAGGACACACTGAGG	GATCCACTGAGGCAGGCACATG
EVM0012836	TCCCACCTTCTCGTCAGTCT	CCGACTTTGGAGGACATCACC
EVM0009749	CCGTCACAGCTTGATCCAGTCG	TGTGCCGCCGCTAGTCCTG
EVM0001451	ACCAGAACAAGCCAGCGACTTC	CTGCACGGATGCTGTCTGGATAG
EVM0015987	TCCAATCTCAACACTTGCGGCTAC	GAGACACCGGCTGAGCACAAC
EVM0023113	CACCCACGTCCACGACACATG	ACCGAGGGAGGAGTGTGATGTC

**Table 2:** Statistics of RNA-seq data.

Sample Name	OSIMT1	OSIMT2	OSIMT3	OSIFO1	OSIFO2	OSIFO3
Clean reads	45,015,696	50,841,250	44,986,040	61,504,798	66,830,622	51,194,240
Clean bases	6.72	7.59	6.71	9.19	9.97	7.65
GC content (%)	40.39%	40.53%	40.15%	41.30%	41.29%	39.17%
≥Q30 (%)	93.23%	92.61%	92.94%	93.39%	93.52%	93.65%

**Table 3:** The GO terms related to gonadal development.

GO ID	Terms	Gene ID	Pfam Annotation	Expression Profile
GO:0007548	sex differentiation	EVM0011468	DM DNA binding domain	+
		EVM0021683	Actin	+
		EVM0026626	Forkhead domain	-
GO:0045137	development of primary sexual characteristics	EVM0021683	Actin	+
		EVM0026626	Forkhead domain	-
GO:0019953	sexual reproduction	EVM0010793	Cytochrome P450	+
		EVM0015987	Helix-loop-helix DNA-binding domain	+
		EVM0025062	SprT-like family	+
		EVM0001451	Nucleoside diphosphate kinase; Dpy-30 motif	-
		EVM0007741	Ca binding region; EF-hand domain; Ca2+ insensitive EF hand	-
		EVM0015963	14-3-3 protein	-
GO:0018992	germ-line sex determination	EVM0026626	Forkhead domain	-
GO:0046546	development of primary male sexual characteristics	EVM0026626	Forkhead domain	-
GO:0030238	male sex determination	EVM0026626	Forkhead domain	-
GO:0007542	primary sex determination, germ-line	EVM0026626	Forkhead domain	-
GO:0007530	sex determination	EVM0026626	Forkhead domain	-
GO:0007538	primary sex determination	EVM0026626	Forkhead domain	-
GO:0019100	male germ-line sex determination	EVM0026626	Forkhead domain	-
GO:0046661	male sex differentiation	EVM0026626	Forkhead domain	-

Note: “+” means up-regulated; “-”means down-regulated.

**Table 4:** Sex-related DEGs selected from the gonadal transcriptome data of *O. sinensis*.

Gene Name	Gene ID	Average FPKM		Functional Annotation	FDR
		Male	Female		
*Dmrt1*	EVM0011468	136.43	1.56	doublesex- and mab-3-related transcription factor 1	E 3.48 × 10^−5^
*FoxN5*	EVM0026626	97.31	11.58	forkhead box protein N-5-like	1.14 × 10^−15^
*Foxj1*	EVM0024813	65.67	3.77	forkhead box protein J1-B-like	2.18 × 10^−15^
*Sox14*	EVM0023113	0.01	142.93	transcription factor Sox-14-like	2.20 × 10^−47^
*Sox30*	EVM0007650	66.79	0.40	transcription factor Sox-30-like	2.73 × 10^−23^
*Fem1*	EVM0010330	18.17	3.29	Sex-determining protein fem-1	5.17 × 10^−7^
*Pgk2*	EVM0010209	9063.40	195.61	phosphoglycerate kinase 2-like	1.00 × 10^−3^
*Pde*	EVM0008366	13.93	0	cGMP-specific 3′,5′-cyclic phosphodiesterase-like	8.93 × 10^−10^
*Tssk6*	EVM0009652	89.66	0.01	testis-specific serine/threonine-protein kinase 6-like	1.44 × 10^−21^
*Tssk1*	EVM0002859	5.38	0	testis-specific serine/threonine-protein kinase 1-like	9.00 × 10^−3^
*Tssk3*	EVM0024515	395.24	0.02	testis-specific serine/threonine-protein kinase 3-like	9.30 × 10^−18^
*Wdy*	EVM0011883	50.65	6.23	WD repeat-containing protein on Y chromosome	9.21 × 10^−7^
*Lrrc34*	EVM0020929	101.76	0.16	leucine-rich repeat-containing protein 34-like	7.25 × 10^−15^
*Nanos3*	EVM0001816	2.81	66.13	protein nanos 3	1.18 × 10^−9^
*Hsp70*	EVM0023738	12.99	0.57	heat shock protein 70 B2-like	3.18 × 10^−15^
*β-tubulin*	EVM0022450	85.13	3211.12	tubulin beta chain-like	8.62 × 10^−8^
*Pabpc1*	EVM0003110	0.36	829.37	polyadenylate-binding protein	9.46 × 10^−46^
*Col4a5*	EVM0025286	0.12	39.64	collagen alpha-5(IV) chain-like	3.99 × 10^−10^
*Col4a2*	EVM0024483	10.92	130.40	collagen alpha-2(IV) chain-like	4.37 × 10^−7^
*Dnmt1*	newGene_40535	0.73	8.71	DNA (cytosine-5)-methyltransferase 1-like	5.40 × 10^−10^
*Suh*	EVM0000797	1.24	10.22	recombining binding protein suppressor of hairless-like	4.73 × 10^−13^

## Data Availability

All sequencing data associated with this project were deposited in the National Center for Biotechnology Information (NCBI) Sequence Read Archive database with the accession number SRP499429 under the Bioproject PRJNA1095235 (accessed on 3 April 2024, https://www.ncbi.nlm.nih.gov/sra/PRJNA1095235).

## Supplementary Materials

The following supporting information can be downloaded at https://www.mdpi.com/article/10.3390/genes15060682/s1: Table S1: Statistics of clean reads mapped to the reference genome; Table S2: Statistics of annotated new genes; Table S3: Functional annotation of DEGs between testis and ovary of *O. sinensis*; Table S4: The genes included in the wnt signaling pathways of *O. sinensis* and their annotations; Table S5: The genes included in the TGF-β signaling pathways of *O. sinensis* and their annotations; Table S6: The genes included in the Notch signaling pathways of *O. sinensis* and their annotations; Table S7: The statistics of GO enrichment of the DEGs in testis.
