# Supplementary material for: Gonadal Transcriptome Sequencing Analysis Reveals the Candidate Sex-Related Genes and Signaling Pathways in the East Asian Common Octopus, Octopus sinensis
Source: Genes (Basel). 2024 May 24;15(6):682. doi: 10.3390/genes15060682 (PMC11202624; doi:10.3390/genes15060682)
Supplement: Supplementary file 1 [file genes-15-00682-s001.zip › genes-2984609-supplementary/supplementary File/Supplementary Table S1.docx]

**Table S1**. Statistics of clean reads mapped to the reference genome

| **Sample name** | **OSIMT1** | **OSIMT2** | **OSIMT3** | **OSIFO1** | **OSIFO2** | **OSIFO3** |
| --- | --- | --- | --- | --- | --- | --- |
| Maped to reference genome |  |  |  |  |  |  |
| Mapped Reads | 41,856,883  (92.98%) | 47,116,323  (92.67%) | 41,546,433  (92.35%) | 57,439,795  (93.39%) | 62,552,327  (93.60%) | 48,335,480  (94.42%) |
| Multiple Map Reads | 1,258,487  (2.80%) | 1,507,130  (2.96%) | 1,325,351  (2.95%) | 1,985,212  (3.23%) | 2,319,700  (3.47%) | 2,025,174  (3.96%) |
| Uniq Mapped Reads | 40,598,396  (90.19%) | 45,609,193  (89.71%) | 40,221,082  (89.41%) | 55,454,583  (90.16%) | 60,232,627  (90.13%) | 46,310,306  (90.46%) |
| Reads Map to '+' | 21,352,814  (47.43%) | 24,080,615  (47.36%) | 21,212,592  (47.15%) | 29,500,342  (47.96%) | 32,253,456  (48.26%) | 24,634,401  (48.12%) |
| Reads Map to '-' | 21,386,192  (47.51%) | 24,138,205  (47.48%) | 21,248,771  (47.23%) | 29,469,487  (47.91%) | 32,216,914  (48.21%) | 24,463,561  (47.79%) |
| Distribution of mapped reads |  |  |  |  |  |  |
| Exon | 68.75% | 65.51% | 63.68% | 75.78% | 75.41% | 75.70% |
| Intron | 5.96% | 5.53% | 5.95% | 5.32% | 5.24% | 4.32% |
| Intergenic | 25.29% | 28.96% | 30.36% | 18.90% | 19.35% | 19.98% |

Note：The value in brackets was the ratio of comparison(%)
