# Supplementary material for: Gonadal Transcriptome Sequencing Analysis Reveals the Candidate Sex-Related Genes and Signaling Pathways in the East Asian Common Octopus, Octopus sinensis
Source: Genes (Basel). 2024 May 24;15(6):682. doi: 10.3390/genes15060682 (PMC11202624; doi:10.3390/genes15060682)
Supplement: Supplementary file 1 [file genes-15-00682-s001.zip › genes-2984609-supplementary/supplementary File/Supplementary Table S2.docx]

**Table S2.** Statistics of annotated new genes

| **Annotated databases** | **New Gene Number** |
| --- | --- |
| COG | 367 |
| GO | 3,097 |
| KEGG | 810 |
| KOG | 834 |
| Pfam | 1,124 |
| Swiss-Prot | 974 |
| eggNOG | 2,163 |
| nr | 5,543 |
| All | 5,567 |
