# Supplementary material for: Gonadal Transcriptome Sequencing Analysis Reveals the Candidate Sex-Related Genes and Signaling Pathways in the East Asian Common Octopus, Octopus sinensis
Source: Genes (Basel). 2024 May 24;15(6):682. doi: 10.3390/genes15060682 (PMC11202624; doi:10.3390/genes15060682)
Supplement: Supplementary file 1 [file genes-15-00682-s001.zip › genes-2984609-supplementary/supplementary File/Supplementary Table S3.docx]

**Table S3**. Functional annotation of DEGs between testis and ovary of *O. sinensis*

| **Functional database** | **Number** |
| --- | --- |
| COG | 2,015 |
| GO | 4,846 |
| KEGG | 2,999 |
| KOG | 3,828 |
| NR | 7,460 |
| Pfam | 4,673 |
| Swiss-Prot | 3,512 |
| eggNOG | 5,370 |
| Functional annotated DEGs (Ratio (%)) | 7,471 (90%) |
