# Supplementary material for: Gonadal Transcriptome Sequencing Analysis Reveals the Candidate Sex-Related Genes and Signaling Pathways in the East Asian Common Octopus, Octopus sinensis
Source: Genes (Basel). 2024 May 24;15(6):682. doi: 10.3390/genes15060682 (PMC11202624; doi:10.3390/genes15060682)
Supplement: Supplementary file 1 [file genes-15-00682-s001.zip › genes-2984609-supplementary/supplementary File/Supplementary Table S4.docx]

**Table S4.** The genes included in the wnt signaling pathways of *O. sinensis* and their annotations

| **Gene ID** | **Nr annotation** | **Expression pattern** |
| --- | --- | --- |
| EVM0001010 | PREDICTED: 1-phosphatidylinositol 4,5-bisphosphate phosphodiesterase beta-4-like [Octopus bimaculoides] | + |
| EVM0001065 | hypothetical protein OCBIM_22033378mg [Octopus bimaculoides] | + |
| EVM0003985 | PREDICTED: transcription factor AP-1-like [Octopus bimaculoides] | + |
| EVM0004563 | PREDICTED: pectin acetylesterase 5-like [Octopus bimaculoides] | + |
| EVM0005813 | PREDICTED: ras-related C3 botulinum toxin substrate 2-like [Octopus bimaculoides] | + |
| EVM0007759 | PREDICTED: low-density lipoprotein receptor-related protein 6-like [Octopus bimaculoides] | + |
| EVM0007872 | PREDICTED: serine/threonine-protein kinase NLK-like, partial [Octopus bimaculoides] | + |
| EVM0014462 | PREDICTED: protein Wnt-10b-like [Octopus bimaculoides] | + |
| EVM0016296 | PREDICTED: BMP and activin membrane-bound inhibitor homolog [Octopus bimaculoides] | + |
| EVM0016582 | PREDICTED: protein Wnt-8b-like isoform X2 [Octopus bimaculoides] | + |
| EVM0017523 | PREDICTED: protein prickle-like [Octopus bimaculoides] | + |
| EVM0019581 | PREDICTED: frizzled-4-like [Octopus bimaculoides] | + |
| EVM0020445 | hypothetical protein OCBIM_22018931mg [Octopus bimaculoides] | + |
| EVM0020603 | hypothetical protein LOTGIDRAFT_159871 [Lottia gigantea] | + |
| EVM0021913 | PREDICTED: protein Wnt-6-like [Octopus bimaculoides] | + |
| EVM0023977 | segment polarity protein dishevelled homolog DVL-3-like isoform X3 [Mizuhopecten yessoensis] | + |
| EVM0026174 | PREDICTED: protein Wnt-5b-like [Octopus bimaculoides] | + |
| EVM0027950 | hypothetical protein OCBIM_22005202mg [Octopus bimaculoides] | + |
| EVM0029807 | hypothetical protein OCBIM_22038925mg [Octopus bimaculoides] | + |
| newGene_25676 | hypothetical protein OCBIM_22014103mg [Octopus bimaculoides] | + |
| newGene_25679 | hypothetical protein OCBIM_22016825mg [Octopus bimaculoides] |  |
| EVM0003272 | PREDICTED: ruvB-like 1 [Octopus bimaculoides] | - |
| EVM0010647 | PREDICTED: serine/threonine-protein phosphatase 2B catalytic subunit alpha isoform-like isoform X5 | - |
| EVM0013354 | hypothetical protein OCBIM_22019350mg [Octopus bimaculoides] | - |
| EVM0017346 | PREDICTED: glycogen synthase kinase-3 beta-like isoform X3 [Biomphalaria glabrata] | - |
| EVM0019263 | hypothetical protein OCBIM_22019350mg [Octopus bimaculoides] | - |
| EVM0026309 | hypothetical protein OCBIM_22015479mg, partial [Octopus bimaculoides] | - |

Note: “+” means up-regulated, “-” means down-regualted.
