# Supplementary material for: Gonadal Transcriptome Sequencing Analysis Reveals the Candidate Sex-Related Genes and Signaling Pathways in the East Asian Common Octopus, Octopus sinensis
Source: Genes (Basel). 2024 May 24;15(6):682. doi: 10.3390/genes15060682 (PMC11202624; doi:10.3390/genes15060682)
Supplement: Supplementary file 1 [file genes-15-00682-s001.zip › genes-2984609-supplementary/supplementary File/Supplementary Table S5.docx]

**Table S5.** The genes included in the TGF-β signaling pathways of *O. sinensis* and their annotations

| **Gene ID** | **Nr annotation** | **Expression pattern** |
| --- | --- | --- |
| EVM0003224 | hypothetical protein OCBIM_22034445mg [Octopus bimaculoides] | + |
| EVM0007575 | PREDICTED: activin receptor type-1-like [Octopus bimaculoides] | + |
| EVM0009392 | PREDICTED: homeobox protein AKR-like isoform X2 [Dendroctonus ponderosae] | + |
| EVM0009959 | mothers against decapentaplegic homolog 6-like [Mizuhopecten yessoensis] | + |
| EVM0013438 | PREDICTED: follistatin-like isoform X1 [Octopus bimaculoides] | + |
| EVM0016296 | PREDICTED: BMP and activin membrane-bound inhibitor homolog [Octopus bimaculoides] | + |
| EVM0024956 | PREDICTED: E3 ubiquitin-protein ligase SMURF2-like isoform X2 [Octopus bimaculoides] | + |
| EVM0025341 | PREDICTED: transcription factor E2F5-like [Octopus bimaculoides] | + |
| EVM0026079 | PREDICTED: homeobox protein TGIF2-like isoform X3 [Octopus bimaculoides] | + |
| EVM0026672 | hypothetical protein OCBIM_22030105mg [Octopus bimaculoides] | + |
| EVM0030866 | PREDICTED: transcription factor E2F5-like [Octopus bimaculoides] | + |

Note: “+” means up-regulated, “-” means down-regualted.
