# Supplementary material for: Gonadal Transcriptome Sequencing Analysis Reveals the Candidate Sex-Related Genes and Signaling Pathways in the East Asian Common Octopus, Octopus sinensis
Source: Genes (Basel). 2024 May 24;15(6):682. doi: 10.3390/genes15060682 (PMC11202624; doi:10.3390/genes15060682)
Supplement: Supplementary file 1 [file genes-15-00682-s001.zip › genes-2984609-supplementary/supplementary File/Supplementary Table S6.docx]

**Table S6.** The genes included in the Notch signaling pathways of *O. sinensis* and their annotations

| **Gene ID** | **Nr annotation** | **Expression pattern** |
| --- | --- | --- |
| EVM0000797 | PREDICTED: recombining binding protein suppressor of hairless-like isoform X5 [Octopus bimaculoides] | + |
| EVM0008462 | PREDICTED: neurogenic locus protein delta-like [Octopus bimaculoides] | + |
| EVM0014946 | PREDICTED: fibropellin-1-like isoform X2 [Octopus bimaculoides] | + |
| EVM0015391 | PREDICTED: E3 ubiquitin-protein ligase DTX4-like [Octopus bimaculoides] | + |
| EVM0017101 | PREDICTED: neurogenic locus notch homolog protein 1-like [Octopus bimaculoides] | + |
| EVM0018047 | PREDICTED: fibropellin-1-like isoform X2 [Octopus bimaculoides] | + |
| EVM0020821 | PREDICTED: delta-like protein 1 [Octopus bimaculoides] | + |
| EVM0023977 | segment polarity protein dishevelled homolog DVL-3-like isoform X3 [Mizuhopecten yessoensis] | + |
| EVM0026176 | PREDICTED: fibropellin-1-like isoform X2 [Octopus bimaculoides] | + |
| EVM0028802 | protein hairy-like isoform X2 [Mizuhopecten yessoensis] | + |

Note: “+” means up-regulated, “-” means down-regualted.
